# Dysbiosis of the oral–gut microbiome in PCOS patients and its implication for noninvasive diagnosis

**DOI:** 10.1002/ctm2.70001

**Published:** 2024-08-14

**Authors:** Feiling Huang, Junru Chen, Miao Zhou, Ruiyi Tang, Shenglong Xu, Yan Zhao, Mingming Su, Xinlei Zhang, Peng Zhang, Rong Chen

**Affiliations:** ^1^ Department of Obstetrics and Gynecology Peking Union Medical College Hospital, Chinese Academy of Medical Sciences & Peking Union Medical College National Clinical Research Center for Obstetric & Gynecologic Diseases Beijing China; ^2^ Institute of Chinese Medical Sciences University of Macau Macao China; ^3^ Beijing ClouDNA Technology Co., Ltd. Beijing China; ^4^ Department of Otolaryngology Head and Neck Surgery Beijing Tongren Hospital, Capital Medical University Beijing China; ^5^ Beijing Key Laboratory for Genetics of Birth Defects, Beijing Pediatric Research Institute, MOE Key Laboratory of Major Diseases in Children; Rare Disease Center, Beijing Children’s Hospital Capital Medical University, National Center for Children’s Health Beijing China

Dear Editor,

Polycystic ovary syndrome (PCOS), characterized by ovulatory dysfunction, androgen excess, and polycystic ovaries, is usually accompanied by metabolic disorders such as obesity, dyslipidemia, and insulin resistance.^1^ PCOS affects 5% to 20% of women who are of reproductive age and is one of the most prevalent reproductive endocrine disorders.^2^ The dysbiosis of gut microbiota (DOGMA) theory of PCOS was developed decades ago, suggesting that dysbiosis of the gut microbiota stimulates the immune system, drives up serum insulin levels, disrupts insulin receptor function, increases the level of androgens deprived of ovaries, and interferes with normal follicle development.^3^ However, to our knowledge, few studies have simultaneously evaluated the gut and oral microbiome in PCOS at the same study.

In this present study, we analyzed the oral–gut microbiome dysbiosis in PCOS patients and healthy controls and identified a universal microbiome‐derived signature that can be used to predict PCOS. Forty‐seven patients with PCOS and 20 age‐matched healthy control donors were enrolled, and clinical information was collected. Individuals with PCOS had significantly higher BMI, WHR, percentage of body fat (PBF), serum testosterone level, LH level, fasting insulin level, fasting glucose level and HOMA‐IR value, alanine transaminase level, TG, and HDL‐C level than healthy controls. As expected, PCOS‐related phenotypes were typically recorded among patients with PCOS (Table S1). To further illuminate the characteristics of microbial community structure associated with PCOS, we first calculated the relative abundance of the dominant bacteria (Figure 1A). The faecal microbiota of the PCOS group was characterized by a higher abundance of *Bacteroidetes* at the phylum level and a lower abundance of *Firmicutes* than healthy women and at the genus level, microbiota dysbiosis in PCOS was characterized by a decrease in *Prevotella* and *Faecalibacterium* and an expansion of genera *Blautia* and *Bacteroides*. In the oral microbiota, the abundance of the phylum *Firmicutes*, genera *Neisseria* and *Streptococcus* decreased but phylum *Proteobacteria* and genus *Pseudomonas* increased in PCOS samples. But notably, the differences in the predominant genera across the faecal and oral microbiota were consistent across the different groups (Figure 1B). We next conducted a Venn analysis based on the genus profile to identify genera that were related to community separation among the four compartments (Figure 1C). Alpha diversity was calculated by the observed features and Faith pd (Figure 1D). We found a higher faecal bacterial alpha diversity in PCOS patients and significantly higher saliva bacterial alpha diversity in PCOS patients. However, when comparing the faecal‐ and saliva bacterial alpha diversity, no significant difference was found in both groups. As shown in Figure 1E, the principal coordinate analysis presented a differential clustering pattern across different health statuses and organ sites (PERMANOVA, *p* < 0.01).

**FIGURE 1:**
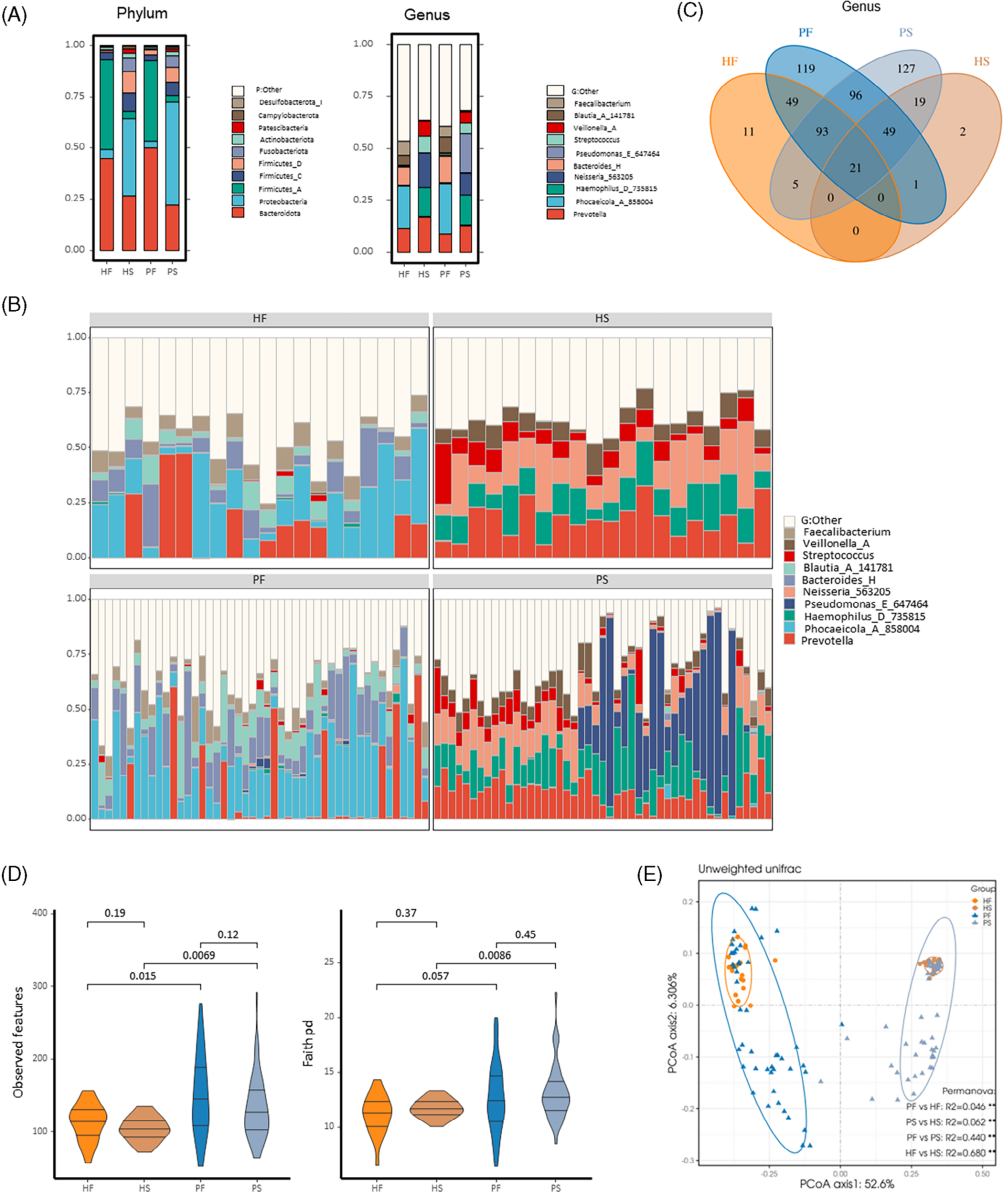
Diversity analysis of the faecal and oral microbiota associated with PCOS. (A) Average relative abundance of prevalent microbiota at the phylum level (left) and genus level (right) in the four compartments. (B) Venn diagram showing the overlap of genera between each compartment. (C) The relative abundances of discriminatory bacteria enriched in each sample. (D) Comparison of alpha diversity of the gut and saliva microbiota using the observed features (left) and the Faith pd (right). (E) PCoA of beta diversity among samples of the four compartments analyzed (*p  <  0.05; **p  <  0.01). HF, faecal samples of healthy controls; HS, saliva samples of healthy controls; PF, faecal samples of patients with PCOS; PS, saliva samples of patients with PCOS.

LEfSe analysis revealed a decrease in genus *Faecalibacterium* and families such as *Ruminococcaceae, Burkholderiaceae*, and *CAG_74* in PCOS patients than healthy controls (Figure 2A). The differences between the oral microbiota from the two groups were also analyzed. PCOS patients exhibited an increase in *Pseudomonadaceae, Pseudomonadales, Pseudomonadas, Proteobacteria*, and *Gammaproteobacteria* (Figure 2A). The heatmap shows the relative abundances of the significant bacterial taxa for each sample (Figure 2B). Gut microbiome functionality based on KEGG pathways revealed significant differences between women with PCOS and healthy controls (*p* < 0.05, Figure 3A). Remarkably, in comparison with healthy controls, the gut microbiota of PCOS patients showed enrichment in tropane, piperidine, and pyridine alkaloid biosynthesis. We found enrichment in taurine and hypotaurine metabolism, cyanoamino acid metabolism, and beta‐Alanine metabolism in women with PCOS (Figure 3B). Taken together, our results showed that not only the microbiome ecological structures but also the functional profiles of the two groups were significantly different. In order to find PCOS‐associated biomarkers, random forest regression models were constructed, and the combination analysis revealed that a group of the key 34 genera provided the best discriminatory power. Next, we examined the associations between the differential genera and clinical factors and we found 19 genera in the gut while 15 genera in the saliva were correlated with clinical factors (Figure 3C,D).

**FIGURE 2:**
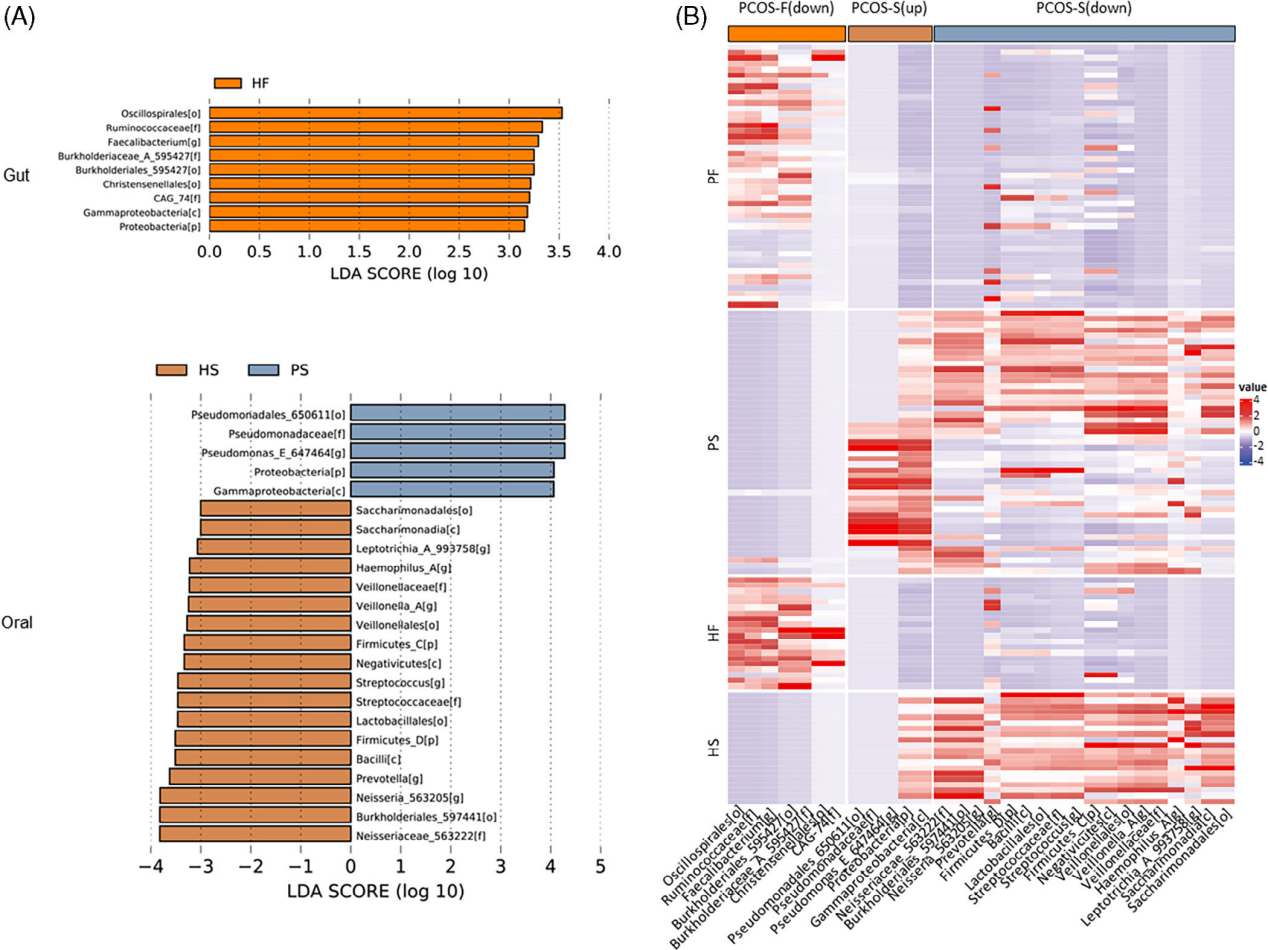
Microflora dysbiosis of the oral–gut axis in patients with PCOS. (A) Results of LEfSe analysis showing bacterial taxa that were significantly different in abundance in the gut (the upper) and oral (the lower) microbiomes between PCOS patients and healthy controls, with an absolute value of log LDA score > 3.0. (B) Heatmap showing the relative abundances of the significant bacterial taxa for each sample. HF, faecal samples of healthy controls; HS, saliva samples of healthy controls; PF, faecal samples of patients with PCOS; PS, saliva samples of patients with PCOS.

**FIGURE 3:**
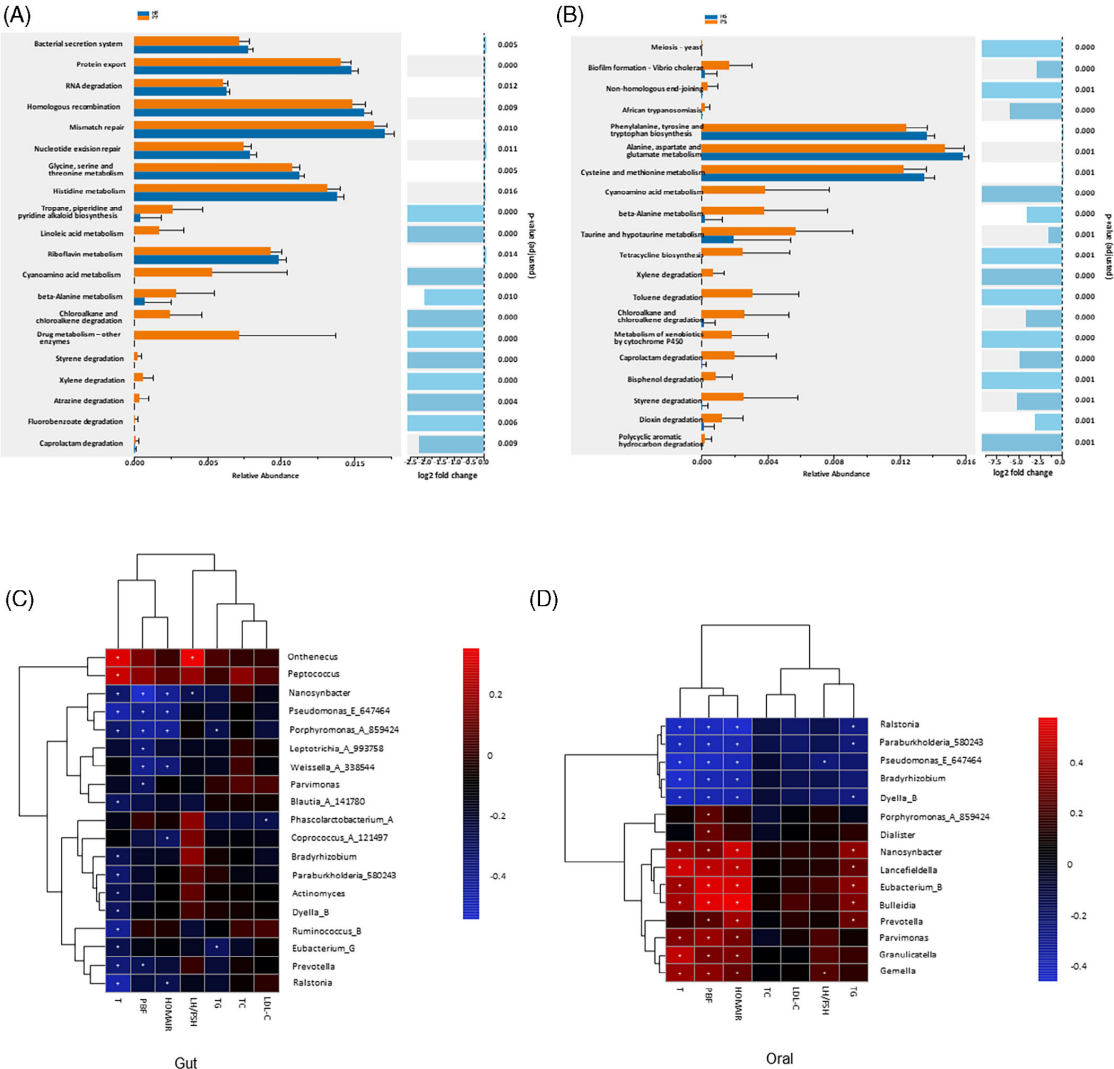
Functional analysis of the faecal and oral microbiota associated with PCOS. (A) Relative abundance and fold change for the significant differential KEGG pathways in faecal microbiota between women with PCOS and healthy controls. (B) Relative abundance and fold change for the significant differential KEGG pathways in oral microbiota between women with PCOS and healthy controls. (C) 19 key bacteria (genus level) in the gut were correlated with clinical. (D) 15 key bacteria (genus level) in the saliva were correlated with clinical (^+^ *p* < 0.01; *p < 0.05). HOMA−IR, insulin resistance index; LDL‐C, low‐density lipoprotein cholesterol; LH/FSH, follicle‐stimulating hormone/luteinizing hormone; PBF, percentage of body fat; T, testosterone; TC, total cholesterol; TG, triglycerides.

The topic of whether the modification of the genus profile may be utilized to distinguish PCOS participants from control subjects was prompted by the notable discrepancy in the oral and gut microbiota composition between individuals with PCOS and healthy controls. To exploit the potential of genera and clinical indices in PCOS identification, random forest classifiers using variables of genera and clinical indices were built, and the area under the ROC curve (AUC) was utilized to evaluate the performance of the classifiers. As shown in Figure 4A, based on the genus profiles of all samples, 11 genera were selected and used as features to construct three random forest classifiers, each based on the randomly chosen training sets from faecal samples, saliva samples, and all samples combined, respectively. The performances of these models were evaluated based on the test sets (Figure 4B). The models utilizing only faecal samples, only saliva samples, and both faecal and saliva samples together demonstrated AUCs of .8, .82, and .73, respectively, indicating moderate discriminatory ability in distinguishing PCOS patients from healthy controls. Moreover, we investigated the utility of the classifier based on 11 genera and 4 clinical indices, namely, PBF, T, LH/FSH, and HOMA−IR. The performance of the classifier was increased after adding four clinical indices (AUC = .83, Figure 4B), indicating the importance of clinical indicators to assist in PCOS diagnosis. Clinical diagnosis of PCOS based on the clinical examination itself has one set or even multiple sets of complex judgment rules. We combined microbial indicators with basic clinical indicators when constructing the random forest model, and these findings suggested that the microbiome may be useful as a predictive marker for PCOS diagnosis and that the combination of clinical indices could optimize the accuracy of PCOS discrimination.

**FIGURE 4:**
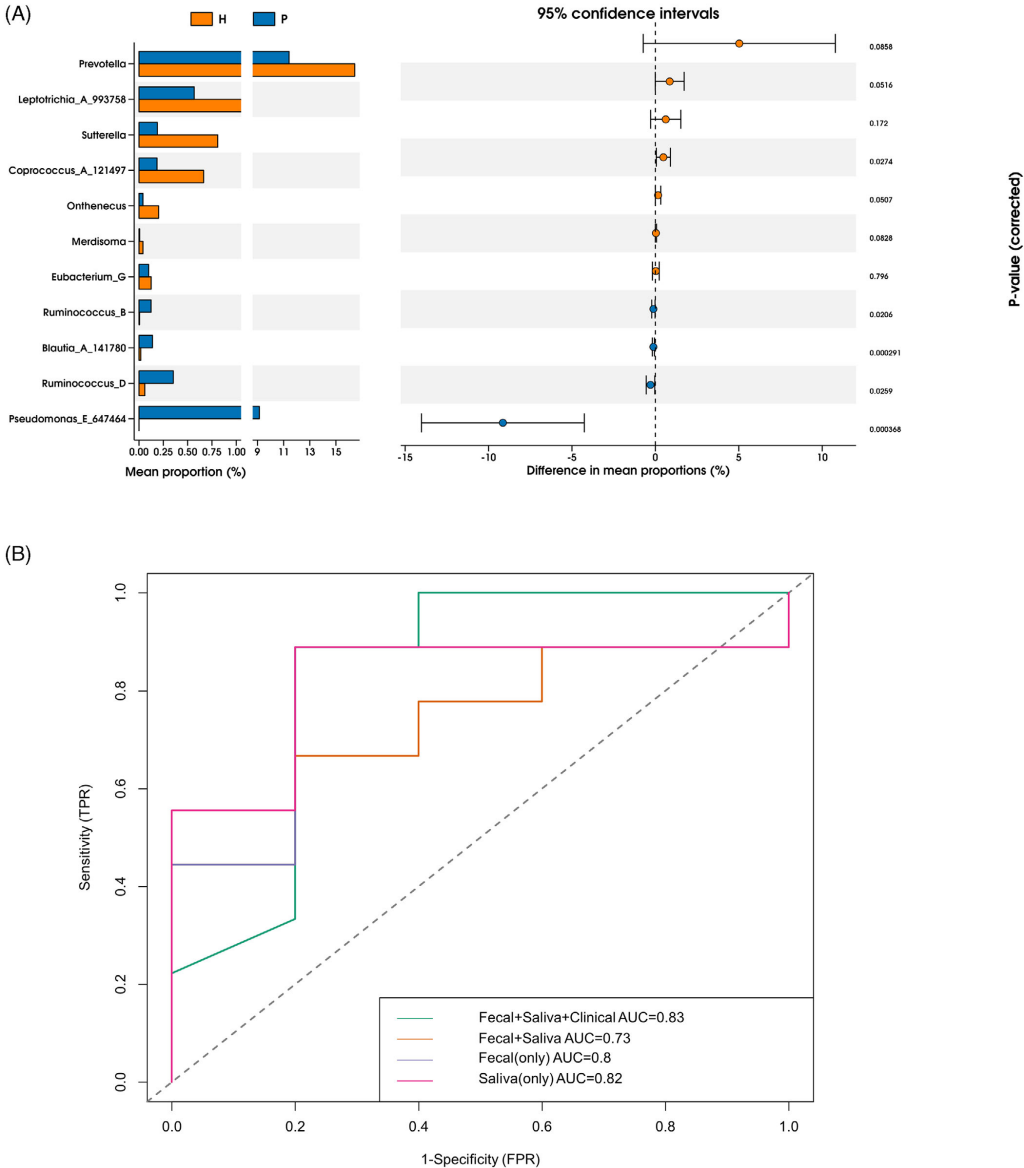
The microbiota and clinical factors could be used to predict PCOS based on random forest models. (A) Bar plot showing coefficients of selected marker genera (left); the 95% confidence intervals of these markers (right). (B) Receiver operating characteristic curves of random forest models were constructed based on the selected genera alone and selected genera plus clinical factors. The AUC displayed in the lower right‐hand corner shows the performance of the models.

These findings suggest that both oral and gut microbiota dysbiosis exist in PCOS patients and these conditions are correlated with the phenotypes of PCOS. Combining faecal and oral microbial indicators shows a better performance of discrimination from health status than using oral microbiota or faecal microbiota alone, and the combination of clinical indices could optimize the accuracy. The oral and gut microbiota probably have the potential as predictive markers in PCOS diagnosis.

## AUTHOR CONTRIBUTIONS

Rong Chen initiated the project and designed the research. Ruiyi Tang, Peng Zhang and Xinlei Zhang supervised the studies and Peng Zhang designed the analysis. Feiling Huang and Junru Chen wrote and Rong Chen and Peng Zhang revised the manuscript. Feiling Huang conducted participants’ enrollment and part of the experiments. Feiling Huang, Miao Zhou and Junru Chen analyzed the data. Shenglong Xu, Yan Zhao and Mingming Su assisted with data analysis, results interpretation, and manuscript editing.

## CONFLICT OF INTEREST STATEMENT

M.Z., M.S., and X.Z. were the employees of Beijing ClouDNA Technology Co., Ltd. The remaining authors declare no conflict of interest.

## FUNDING INFORMATION

This work was supported by the National Natural Science Foundation of China (81871141 and 82201781), the National High‐Level Hospital Clinical Research Funding (2022‐PUMCH‐B‐123), the National Key Research and Development Program (2018YFC1004801), and the CAMS Innovation Fund for Medical Sciences (CIFMS) (2020‐I2M‐CT‐B‐040).

## ETHICS APPROVAL AND CONSENT TO PARTICIPATE

The experimental procedures of this study were approved by the Ethics Review Committee of Peking Union Medical College Hospital Medical (JS‐1691). All participants were recruited from the Peking Union Medical College Hospital between February 2019 and March 2020.

## Supporting information

Supporting Information

Supporting Information

## Data Availability

The processed data and analysis codes are available upon reasonable request from the corresponding author.
