# Supplementary material for: Dysbiosis of the oral–gut microbiome in PCOS patients and its implication for noninvasive diagnosis
Source: Clin Transl Med. 2024 Aug 14;14(8):e70001. doi: 10.1002/ctm2.70001 (PMC11324685; doi:10.1002/ctm2.70001)
Supplement: Supplementary file 1 — Supporting Information [file CTM2-14-e70001-s001.docx]

**Supplementary Table 1. Clinical characteristics of participants**

|  | PCOS  (n=47) | Control  (n=20) | *P* value |
| --- | --- | --- | --- |
| Age (year) | 27.8±4.8 | 27.2±4.66 | 0.623 |
| BMI (kg/m^2^) | 24.9±5.28 | 20.7±1.66 | <0.001 |
| WHR | 0.83±0.07 | 0.79±0.02 | <0.001 |
| Body fat % | 33.5±8.16 | 23.9±4.17 | <0.001 |
| T (ng/ml) | 0.81±0.26 | 0.59±0.11 | <0.001 |
| DHEAS (μg/dl) | 282±95 | 263±111 | 0.528 |
| FSH (IU/L) | 6.27±2.11 | 5.59±2.08 | 0.239 |
| LH (IU/L) | 12.3±6.73 | 7.66±4.82 | 0.003 |
| LH/FSH | 2.05±1.07 | 1.51±0.99 | 0.059 |
| Fasting glucose level (mmol/l) | 5.08±0.83 | 4.70±0.35 | 0.014 |
| Fasting insulin level  (μIU/ml) | 14.9±11.9 | 5.99±2.64 | <0.001 |
| HOMA-IR | 3.57±3.21 | 1.25±0.59 | <0.001 |
| T-25OHD (ng/ml) | 13.0±4.47 | 20.8±33.5 | 0.350 |
| ALT (U/L) | 20.9±14.6 | 11.6±3.96 | <0.001 |
| AST (U/L) | 20.5±9.19 | 19.3±3.48 | 0.455 |
| CR (μmol/L) | 58.5±8.47 | 62.6±8.74 | 0.085 |
| UREA (mmol/l) | 3.91±0.9 | 4.28±0.94 | 0.155 |
| TC (mmol/l) | 4.62±0.65 | 4.51±0.59 | 0.49 |
| TG (mmol/l) | 0.92±0.53 | 0.60±0.26 | 0.002 |
| HDL-C (mmol/l) | 1.46±0.31 | 1.67±0.29 | 0.018 |
| LDL-C (mmol/l) | 2.74±0.66 | 2.47±0.53 | 0.088 |
| Irregular menstruation (%) | 47 (100) | 0 (0) | <0.001 |
| Hirsutism (%) | 17 (36.2) | 0 (0) | 0.001 |
| Acne (%) | 10 (21.3) | 0 (0) | 0.027 |
| PCOM (%) | 44 (93.6) | 0 (0) | <0.001 |

BMI, body mass index; WHR, waist-hip ratio; T, testosterone; DHEAS, dehydroepiandrosterone sulfate; FSH, follicle-stimulating hormone; LH, luteinizing hormone; HOMA-IR, insulin resistance index; TC, total cholesterol; TG, triglycerides; HDL-C, high-density lipoprotein cholesterol; LDL-C, low-density lipoprotein cholesterol; PCOM, polycystic ovary morphology; PCOS, polycystic ovary syndrome. Continuous variables are presented as the mean ± SD and were analyzed by two-tailed Student’s t test (for two groups). Categorical variables are presented as n (%) and were analyzed by Fisher’s test.
