# Supplementary material for: Dysbiosis of the oral–gut microbiome in PCOS patients and its implication for noninvasive diagnosis
Source: Clin Transl Med. 2024 Aug 14;14(8):e70001. doi: 10.1002/ctm2.70001 (PMC11324685; doi:10.1002/ctm2.70001)
Supplement: Supplementary file 2 — Supporting Information [file CTM2-14-e70001-s002.docx]

**Supplementary Materials and Methods**

**Human subjects**

*Inclusion of patients*

The experimental procedures of this study were approved by the Ethics Review Committee of Peking Union Medical College Hospital Medical (JS-1691). All participants were recruited from the Peking Union Medical College Hospital between February 2019 and March 2020. We enrolled 47 patients with PCOS and 20 healthy controls. PCOS diagnosis was based on the 2003 Rotterdam criteria ^1^ with at least two of the following symptoms: (1) oligo-ovulation and/or anovulation; (2) androgen excess, either biochemical or clinical; (3) polycystic ovaries on ultrasound assessment. Those with thyroid disease, hyperprolactinemia, androgen-producing tumors, Cushing syndrome, and nonclassic congenital adrenal hyperplasia were excluded. None of the individuals with PCOS had been treated with PCOS-related therapy. The healthy controls with normal ovarian morphology and regular menstrual cycles, without signs of clinical and/or biochemical hyperandrogenism were recruited from the general community. Those who were pregnant or breastfeeding within a year, who received an antibiotic, Chinese traditional medicine, or oral contraceptive within the past 3 months, who had abnormal thyroid or adrenal function, or who were underweight were excluded. Written informed consent was acquired from all patients and healthy controls.

*Clinical inspection*

Body weight, height, waist and hip circumference were measured; body composition was analyzed, and body mass index (BMI) was calculated as the ratio of body weight (kg) to height (m^2^), and waist-to-hip ratio (WHR) was calculated was calculated as the ratio of waist circumference (cm) to hip circumference (cm). Fasting blood samples were collected on days 2–4 of their spontaneous cycles.

Hormone including luteinizing hormone (LH), follicle-stimulating hormone (FSH), dehydroepiandrosterone sulfate (DHEAS), testosterone and estradiol were tested with an automated chemiluminescence analyzer (Beckman DXI800). The levels of fasting glucose, alanine transaminase, aspartate transaminase, creatinine, urea, creatinine, urea, low-density lipoprotein cholesterol (LDL-C), high-density lipoprotein cholesterol (HDL-C), total cholesterol (TC) and triglycerides (TG) were measured using an autoanalyzer (Abbott c16000). The fasting insulin (FIN) level was measured with a Siemens Atellica system. T-25 hydroxyvitamin D was measured with a Roche Cobas test. The insulin resistance index (HOMA-IR) was calculated as fasting insulin (µIU/ml) × fasting glucose (mmol/l)/22.5, with homeostasis model assessment methods.

*Microbial specimen collection*

Stool and oral specimens were collected according to the Human Microbiome Project (HMP) collection protocol ^2^. Before sample collection, none of the PCOS patients had received any antibiotic treatment within the past 3 months. Stool samples were picked using the Stool Collection Tubes with DNA Stabilizer (1038111200, Invitek). Oral samples were picked using the SalivaGene Collector (1035211200, Invitek). All the oral and stool samples were frozen and stored at -80°C until DNA extraction.

**16S amplicon sequencing and analysis**

*DNA extraction and sequencing*

DNA was extracted using a TIANamp stool DNA kit (DP328, TIANGEN BIOTECH). The concentrations of DNA were then measured with a NanoDrop 1000 (Thermo Fisher Scientific). After assessment of DNA quality and quantification with a Qubit fluorometer (Thermo Fisher Scientific), the V3- V4 region of the 16S rRNA gene was amplified using primers 341F (5’- CCTACGGRRBGCASCAGKVRVGAAT-3’) and 806R (5’-GGACTACNVGGGTWTCTAATCC-3’). Then, after the checking for the size and specificity of PCR products and purifying by agarose gel electrophoresis, the amplicons were sequenced using Illumina MiSeq (Illumina, San Diego, CA, USA).

*16S amplicon analysis*

Paired-end reads of each sample were split from raw data generated by the sequencer through the barcode sequence. These paired-end reads were processed by QIIME2 (version 2023.07) and a series of plugins embedded in QIIME2 ^3^. The cutadapt plugin was used to trim the PCR primers from the paired-end reads ^4^. Both the profile of amplicon sequence variants (ASVs) and representative sequences were obtained via the DADA2 plugin which performed steps of quality filtering, denoising, tags merging, chimera removing and dereplication ^5^. The taxonomic classification of ASVs was assigned via the greengenes2 plugin in QIIME2 and the GreenGenes2 database ^6^.

The within-sample (α) diversity indices and distance matrices of β diversity were calculated by using the diversity plugin in QIIME2, and PCoA was performed and displayed based on the calculated unweighted UniFrac distances by using the stats package in the R software (version 4.2.2). The Wilcoxon rank sum test was undertaken to evaluate between-group differences in α diversity indices. Permutational multivariate analysis of variance (PERMANOVA) was applied with the pairwiseAdonis package (version 0.4.1) in R to compare microbial composition differences between two groups. Linear discriminant analysis effect size (LEfSe) was performed to distinguish significantly different microorganisms between the two groups with a cutoff of |LDA score| > 3. Correlations between genera and clinical indices were calculated by Spearman’s correlation coefficients with the stats package in R. Based on 16S rRNA gene sequencing data, Phylogenetic Investigation of Communities by Reconstruction of Unobserved States (PICRUSt2, version 2.5.2) ^7^ was used to predict functional abundances. Differential abundance analysis of KEGG pathway was performed based on the Maaslin2 method ^8^ in the R package ggpicrust2 (version 1.7.2) ^9^. A *P* value < 0.05 was considered statistically significant

**PCOS prediction model**

Using the profile of genera and clinical indices, samples were randomly divided into training and test sets using the base package (version 4.2.2) in R. In order to evaluate the performance of the predictive model and get the optimum prognostic markers, repeated (10 times) five-fold cross-validation was performed on the training set via the EasyMicroPlot package (version 0.5.1.23) ^10^ in R. Based on those selected prognostic markers, the classification model for predicting PCOS was constructed using the randomForest package (version 4.7.1.1) in R. The ROCit package (version 2.1.1) in R was used to calculate the area under the curve (AUC) to measure the performance of the model and the receiver operating characteristic (ROC) curve was plotted.

**Statistical analysis**

GraphPad Prism (version 9.0) was used for statistical analysis. Kolmogorov–Smirnov normality test was used to determine the sample distribution. For normal distribution, the evaluation of statistical significance between two groups was conducted by two-tailed Student’s t-test. For non-parametric distributions, the evaluation of statistical significance between two groups was conducted by two-tailed Mann–Whitney U-test. Data are shown as mean ± standard deviation, and *P*<0.05 was considered statistically significant.

**References**

1 Revised 2003 consensus on diagnostic criteria and long-term health risks related to polycystic ovary syndrome (PCOS). *Human reproduction (Oxford, England)* **19**, 41-47, doi:10.1093/humrep/deh098 (2004).

2 McInnes, P. & Cutting, M. *Core microbiome sampling protocol A HMP Protocol # 07–001*. Vol. 11 1-109 (2010).

3 Bolyen, E. *et al.* Reproducible, interactive, scalable and extensible microbiome data science using QIIME 2. *Nature biotechnology* **37**, 852-857, doi:10.1038/s41587-019-0209-9 (2019).

4 Kechin, A., Boyarskikh, U., Kel, A. & Filipenko, M. cutPrimers: A New Tool for Accurate Cutting of Primers from Reads of Targeted Next Generation Sequencing. *Journal of computational biology : a journal of computational molecular cell biology* **24**, 1138-1143, doi:10.1089/cmb.2017.0096 (2017).

5 Callahan, B. J. *et al.* DADA2: High-resolution sample inference from Illumina amplicon data. *Nature methods* **13**, 581-583, doi:10.1038/nmeth.3869 (2016).

6 McDonald, D. *et al.* Greengenes2 enables a shared data universe for microbiome studies. 2022.2012. 2019.520774 (2022).

7 Douglas, G. M. *et al.* PICRUSt2 for prediction of metagenome functions. *Nature biotechnology* **38**, 685-688, doi:10.1038/s41587-020-0548-6 (2020).

8 Mallick, H. *et al.* Multivariable association discovery in population-scale meta-omics studies. *PLoS computational biology* **17**, e1009442, doi:10.1371/journal.pcbi.1009442 (2021).

9 Yang, C. *et al.* ggpicrust2: an R package for PICRUSt2 predicted functional profile analysis and visualization. *Bioinformatics (Oxford, England)* **39**, doi:10.1093/bioinformatics/btad470 (2023).

10 Liu, B. *et al.* EasyMicroPlot: An Efficient and Convenient R Package in Microbiome Downstream Analysis and Visualization for Clinical Study. *Frontiers in genetics* **12**, 803627, doi:10.3389/fgene.2021.803627 (2021).
